# Supplementary material for: Classification of botnet attacks in IoT smart factory using honeypot combined with machine learning
Source: PeerJ Comput Sci. 2021 Jan 25;7:e350. doi: 10.7717/peerj-cs.350 (PMC7924422; doi:10.7717/peerj-cs.350)
Supplement: Supplemental Information 1 [file peerj-cs-07-350-s001.docx]

library(caret)

library(dplyr)

library(readr)

#Combine 4 data files into one dataframe

Leedata2 <- list.files(path="D:/Work/TaylorsU/Lee Master Thesis/Dataset/Traning and Testing Tets (5% of the entier dataset)/All features", full.names = TRUE) %>%

lapply(read_csv) %>%

bind_rows

#Extract 10 features

Leedata3 <- select(Leedata2, c(flgs_number,srate,drate,rate,max,state_number,mean,min,stddev,seq,category))

#Category change to factor for classification

Leedata3$category <- as.factor(Leedata3$category)

#Static summary of dataframe

summary(Leedata3)

#Get data type of dataframe

apply(Leedata3, class)

#Extract 100 DDoS, 100 Dos, 100 Reconnaissance, and 76 Theft data

LeeDDos <- subset(Leedata3,category=="DDoS")

LeeDDos <- sample_n(LeeDDos,100)

LeeDos <- subset(Leedata3,category=="DoS")

LeeDos <- sample_n(LeeDos,100)

LeeRecon <- subset(Leedata3,category=="Reconnaissance")

LeeRecon <- sample_n(LeeRecon,100)

LeeTheft <- subset(Leedata3,category=="Theft")

Leedata4 <- rbind(LeeDDos,LeeDos,LeeRecon,LeeTheft)

#Remove normal factor level

Leedata5 <- droplevels(Leedata4)

#export out dataset

write.csv(Leedata5,"D:/Work/TaylorsU/Lee Master Thesis/Dataset/Leedata5.csv",row.names=TRUE)

#split data to 80:20 ratio

LeeTrain <- createDataPartition(Leedata5$category, p=0.8, list=FALSE, times = 1)

Leetraining <- Leedata5[ LeeTrain, ]

Leetesting <- Leedata5[ -LeeTrain, ]

#Random Forest

library("randomForest")

Leemod_RF <- train(category ~ ., data=Leetraining, method="rf")

Leepred_RF = predict(Leemod_RF, newdata=Leetesting)

confusionMatrix(Leepred_RF,Leetesting$category)

# Random committee

library("kernlab")

Leemod_ Random committee <- train(category ~ ., data=Leetraining, method=" Random committee Linear")

Leepred_ Random committee = predict(Leemod_ Random committee, newdata=Leetesting)

confusionMatrix(Leepred_ Random committee Leetesting$category)
