# Supplementary material for: Classification of botnet attacks in IoT smart factory using honeypot combined with machine learning
Source: PeerJ Comput Sci. 2021 Jan 25;7:e350. doi: 10.7717/peerj-cs.350 (PMC7924422; doi:10.7717/peerj-cs.350)
Supplement: Supplemental Information 2 [file peerj-cs-07-350-s002.docx]

**R Code**

#Discretization

library(funModeling)

d_bins=discretize_get_bins(data= simple_dataset_10feature, input=c("flgs_number","srate","drate","rate","max","state_number","mean","min","stddev","seq"),

n_bins=5)

# Checking `d_bins` object:

d_bins

# Now it can be applied on the same data frame or in a new one

Leedata_discretized=discretize_df(data= simple_dataset_10feature, data_bins=d_bins,

stringsAsFactors=T)

View(Leedata_discretized)

sapply(Leedata_discretized, class)

LeeTrain <- createDataPartition(Leedata_discretized$category, p=0.8, list=FALSE, times = 1)

Leetraining <- Leedata_discretized[ LeeTrain, ]

Leetesting <- Leedata_discretized[ -LeeTrain, ]

#Random Forest

library("randomForest")

Leemod_RF <- train(category ~ ., data=Leetraining, method="rf")

Leepred_RF = predict(Leemod_RF, newdata=Leetesting)

confusionMatrix(Leepred_RF,Leetesting$category)

# Random committee

library("kernlab")

Leemod_ Random committee <- train(category ~ ., data=Leetraining, method=" Random committee Linear")

Leepred_ Random committee = predict(Leemod_ Random committee, newdata=Leetesting)

confusionMatrix(Leepred_ Random committee,Leetesting$category)
